# Establishing priorities for a Mental Health strategy in Castilla y Leon: The cohesion of professionals and society

**DOI:** 10.1192/j.eurpsy.2023.1142

**Published:** 2023-07-19

**Authors:** J. M. Pelayo-Terán, Y. Zapico-Merayo, A. M. Saez Aguado, R. Villa Carcedo, Á. Álvaro Prieto

**Affiliations:** 1 Psiquiatría y Salud Mental. Unidad de Calidad y Seguridad del Paciente, Hospital El Bierzo. GASBI. SACYL. CIBERSAM; 2 Área de Medicina Preventiva y Salud Pública. Departamento de Ciencias Biomédicas., Universidad de León; 3 Psiquiatría y Salud Mental, Hospital El Bierzo. GASBI. SACYL, Ponferrada (León); 4 Junta de Castilla y Leon; 5 Servicio de Coordinación, Sociosanitaria y Salud Mental Y SALUD MENTAL; 6 Servicio de Coordinación, Sociosanitaria y Salud Mental Y SALUD MENTAL, Gerencia Regional de Salud de Castilla y León, Valladolid, Spain

## Abstract

**Introduction:**

The reform of mental health care is a key health policy target. Mental health care provision in Spain is designed with national and regional strategies that stablish the objectives to develop. The Castilla y Leon regional strategy 2022-2026 aim to stabilsh the priorities for objectives and actions with stakeholders from th eregional society.

**Objectives:**

To evaluate priorities in the implementation of a Mental Health strategy with the consensus of professionals and society.

**Methods:**

An initial consensus was achieved with the regional health goverment and local mental health representatives, considering the 2022-2026 national strategy and other mental health plans from nearby regions. Lines in the strategy included transversal lines (part of all the mental health scope) and action lines (priorities focused in one relevant field)

Priorities were stablished by different representatives from mental health and other healthcare professionals, social and educational stakeholders, scientific societies, people with mental health disorders and families. After agreeing to participate in the process, they had to answer an online survey. For each line, they have to score from 0 to 10.

**Results:**

500 subjects participated (44% Healthcare workers, 5.8% education or social services, 3.8% Justice, 8,6% workers for associations, 14% Mental Health Care users). All the lines were highly appreciated (mean score >7). Within the transversal lines, the highest score was for the Humanization line (8.81±1.43) and the lowest for the Digitalization line (7.18±1.92). In the Action Lines, the highest score was for Suicide (9.03±11.5) and the lowest for Elder people (8.04±1.94).

Prevention line had higher scores by Education, Justice, Associations and Healthcare professionals and the lowest was for users (F: 2.754; p=0.012). In the Digitalization line the higher scores were in the health professionals and scientific societies and the lowest in the users (F:4.665; p<0.001). In the research, innovation and Training line, the higher scores were for professionals, societies and users and the lowest in the education and justice groups. The only differences found in the Action lines was for the Addiction line, with higher scores for societies, social services, professionals and users and lower in Associations and Justice (F:2.219; p=0,040)

**Image:**

**Figure:**
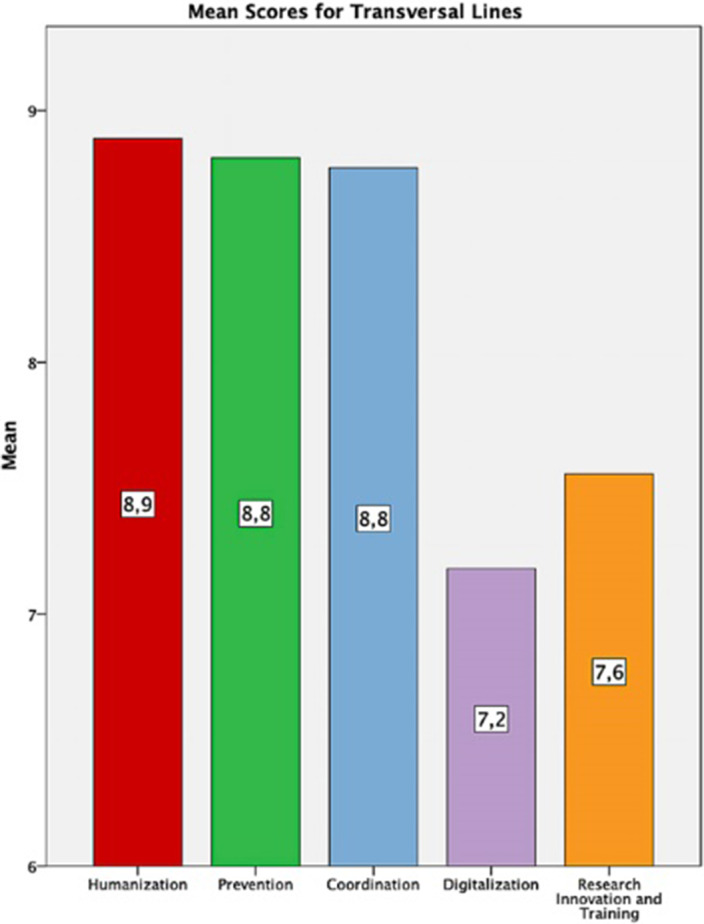

**Image 2:**

**Figure:**
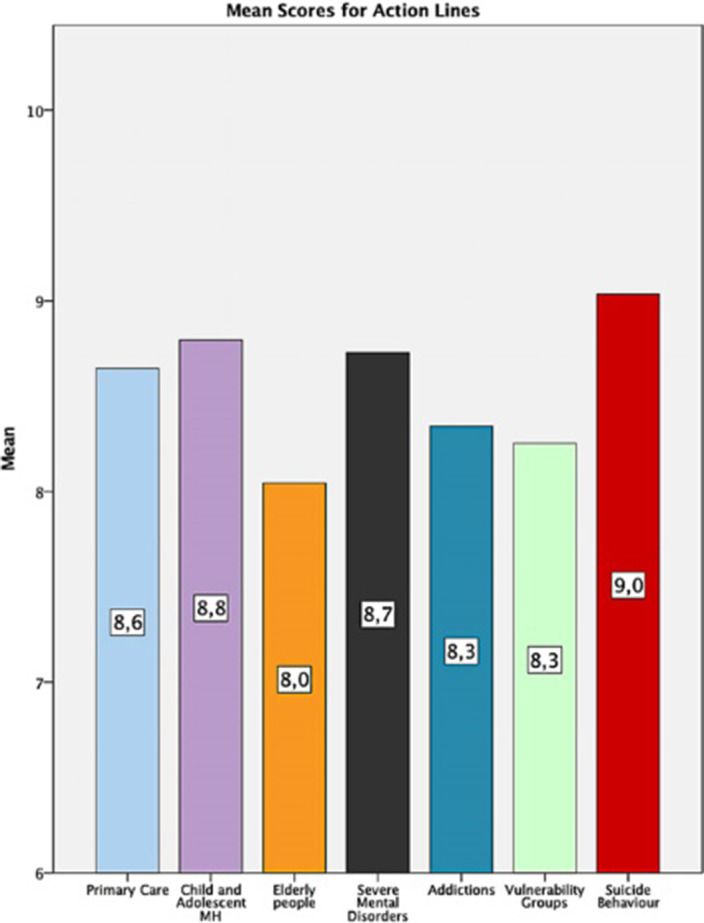

**Conclusions:**

Highest transversal priority for the MH Strategy was Humanization of Mental Health Services, and the most critical action was Suicide prevention. Professionals, Scientific societies and Users considered more important research, innovation and training compared with other society groups, whereas the less important areas for the users were digitalization and prevention users. These priorities will help to design the implementation and schedule for the lines of the Mental Health Strategy in Castilla y León.

**Disclosure of Interest:**

None Declared

